# Supplementary material for: A Rare Benzothiazole Glucoside as a Derivative of ‘Albedo Bluing’ Substance in Citrus Fruit and Its Antioxidant Activity
Source: Molecules. 2024 Jan 6;29(2):302. doi: 10.3390/molecules29020302 (PMC10820657; doi:10.3390/molecules29020302)
Supplement: Supplementary file 1 [file molecules-29-00302-s001.zip › molecules-2774771-supplementary.pdf]

Supporting information

Figure S1. Extract (A) and corresponding precipitate (B) from ‘Gonggan.

Figure S2. Color changes of ABS under different pH values.

Figure S3. The ^{1}H NMR (600 MHz, CD_3OD) spectrum of ABS.

Figure S4. The UV-Vis spectrum of ABS-D.

Figure S5. The MS (A) and MS^2 (B) of ABS-D.

Figure S6. The MS (A) and MS^2 (B) of ABS.

Figure S7. The ^{1}H NMR (600 MHz, $CD_3OD:CF_3COOD=20:1$) spectrum of ABS-D.

Figure S8. The ^{1}H NMR (600 MHz, DMSO-d_6) spectrum of ABS-D.

Figure S9. The ^{13}C NMR (150 MHz, $CD_3OD:CF_3COOD=20:1$) spectrum of ABS-D.

Figure S10. The ^{13}C NMR (150 MHz, DMSO-d_6) spectrum of ABS-D.

Figure S11. The ^{1}H-^{1}H COSY spectrum of ABS-D in $CD_3OD:CF_3COOD=20:1$.

Figure S12. The HSQC spectrum of ABS-D in DMSO-d_6.

Figure S13. The ROESY spectrum of ABS-D in DMSO-d_6.

Figure S14. The HMBC spectrum of ABS-D in DMSO-d_6.

Figure S15. Experimental and calculated chemical shifts for DP4+ probability analysis for three possible isomers of ABS-D.

Table S1. ^{1}H NMR (600 MHz) and ^{13}C NMR (150 MHz) spectra of ABS-D in $CD_3OD:CF_3COOD=20:1$.

Table S2. Calculated and experimental values of ABS-D ^{13}C NMR chemical shifts.

Table S3. B3-LYP geometries for all the optimized compounds.

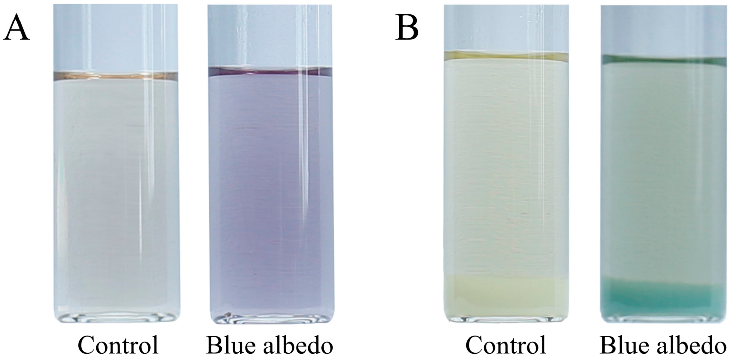

Figure S1. Extract (A) and corresponding precipitate (B) from ‘Gonggan’

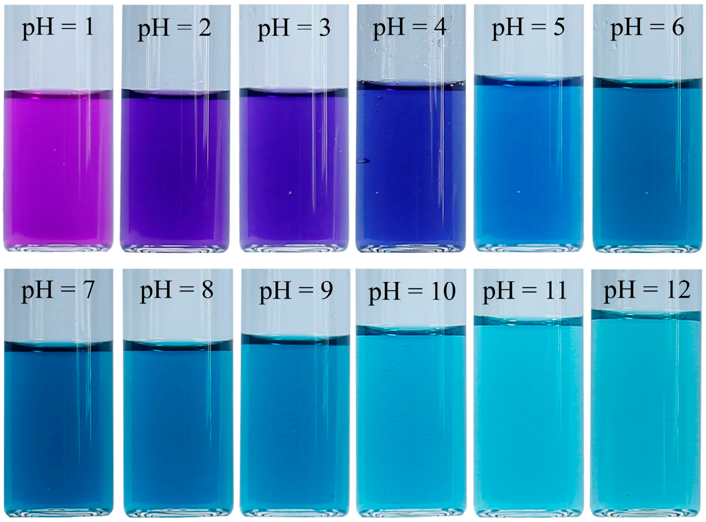

Figure S2. Color changes of ABS under different pH values

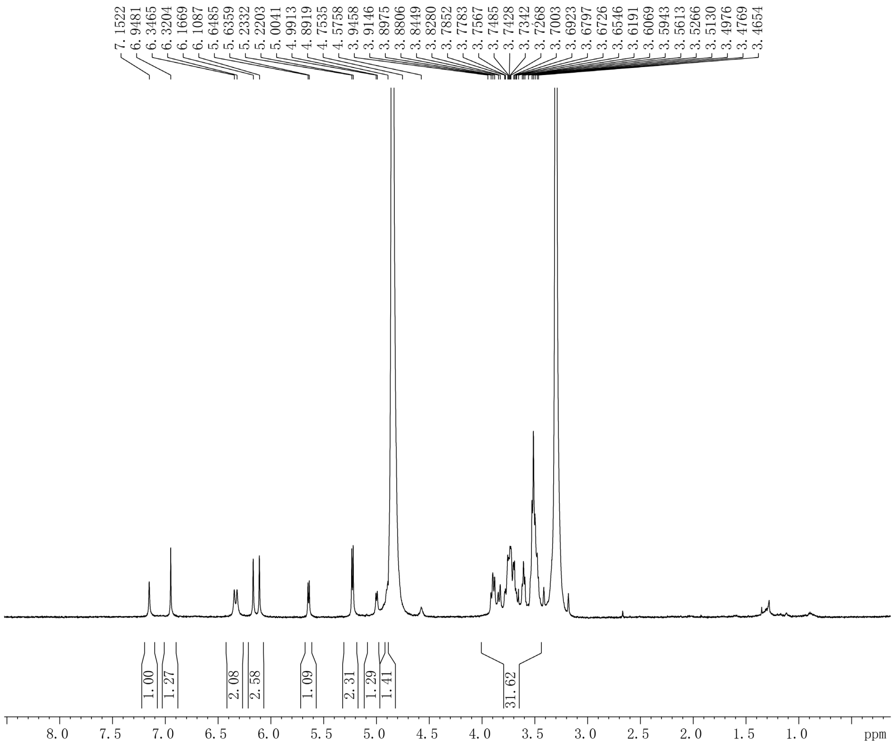

Figure S3 The ^{1}H NMR (600 MHz, CD_3OD) spectrum of ABS

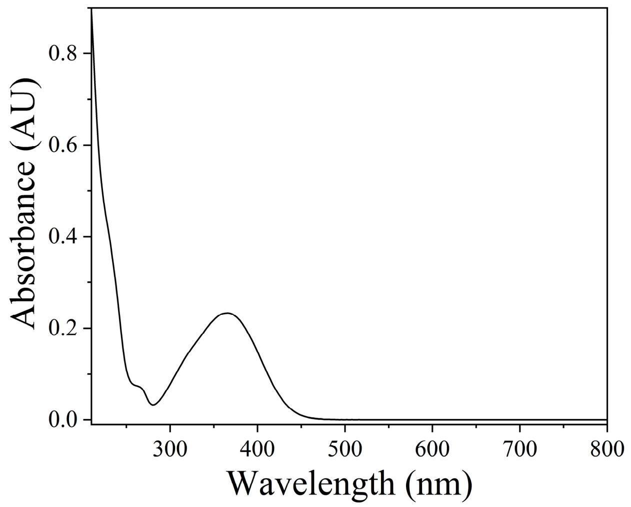

Figure S4 The UV-Vis spectrum of ABS-D.

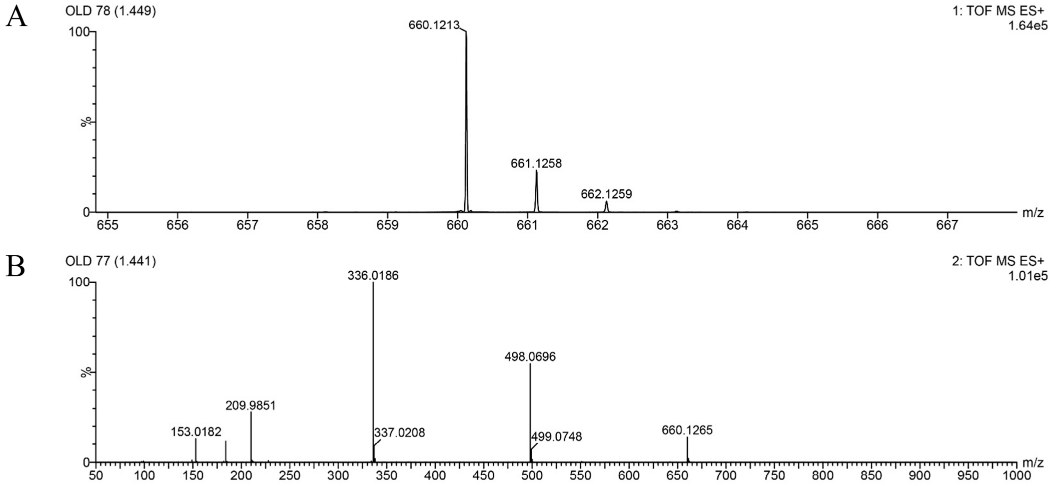

Figure S5 The MS (A) and MS^2 (B) of ABS-D

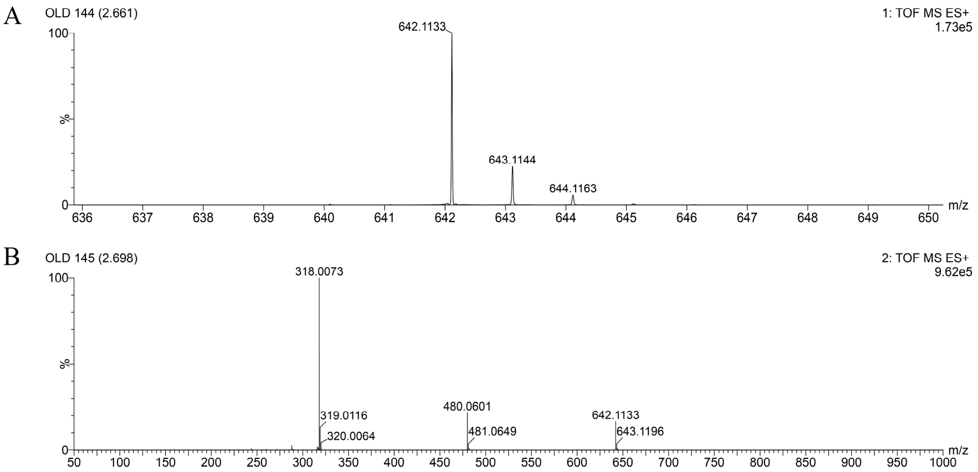

Figure S6 The MS (A) and MS^2 (B) of ABS

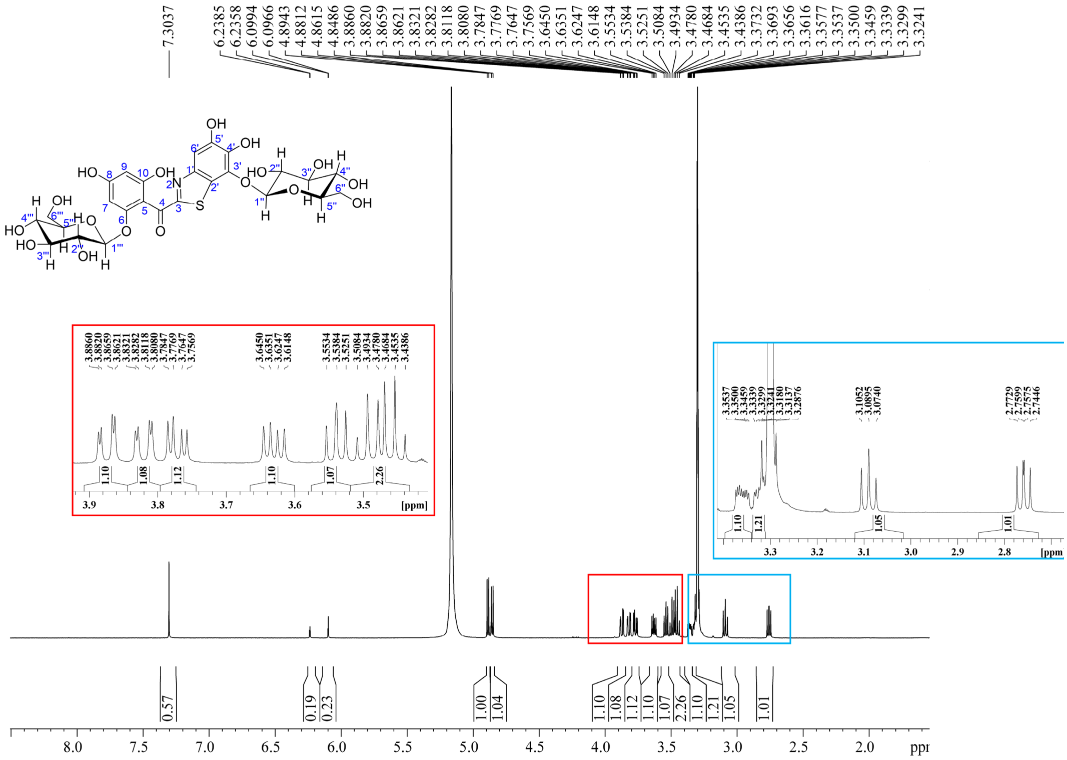

Figure S7. The ^{1}H NMR (600 MHz, CD_3OD:CF_3COOD=20:1) spectrum of ABS-D.

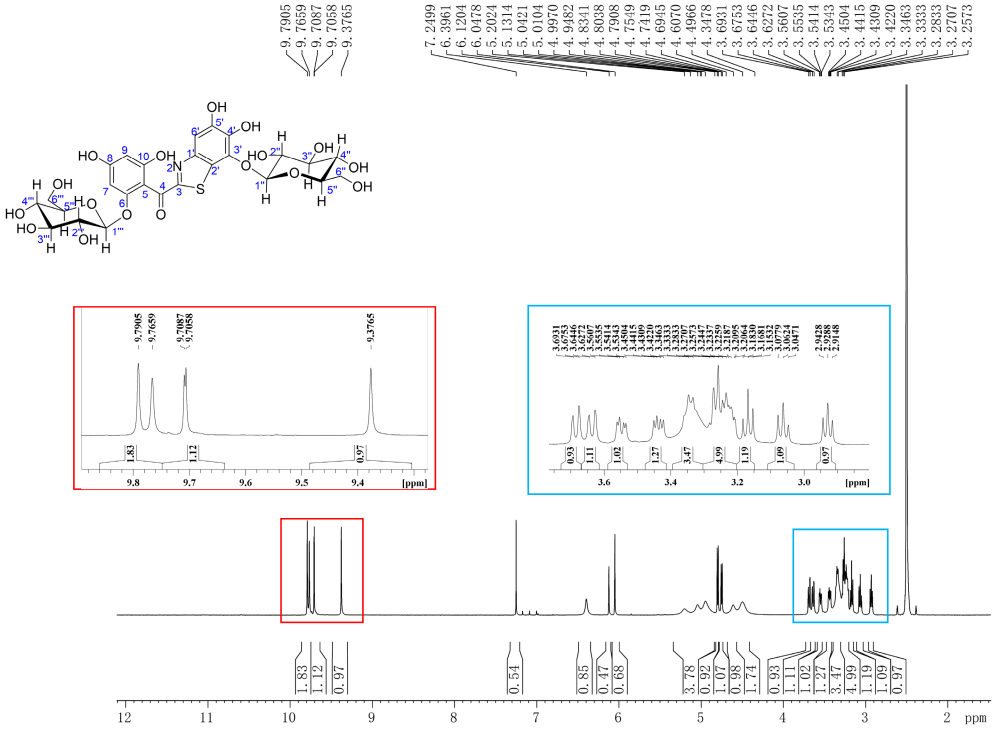

Figure S8. The ^{1}H NMR (600 MHz, DMSO-d_6) spectrum of ABS-D.

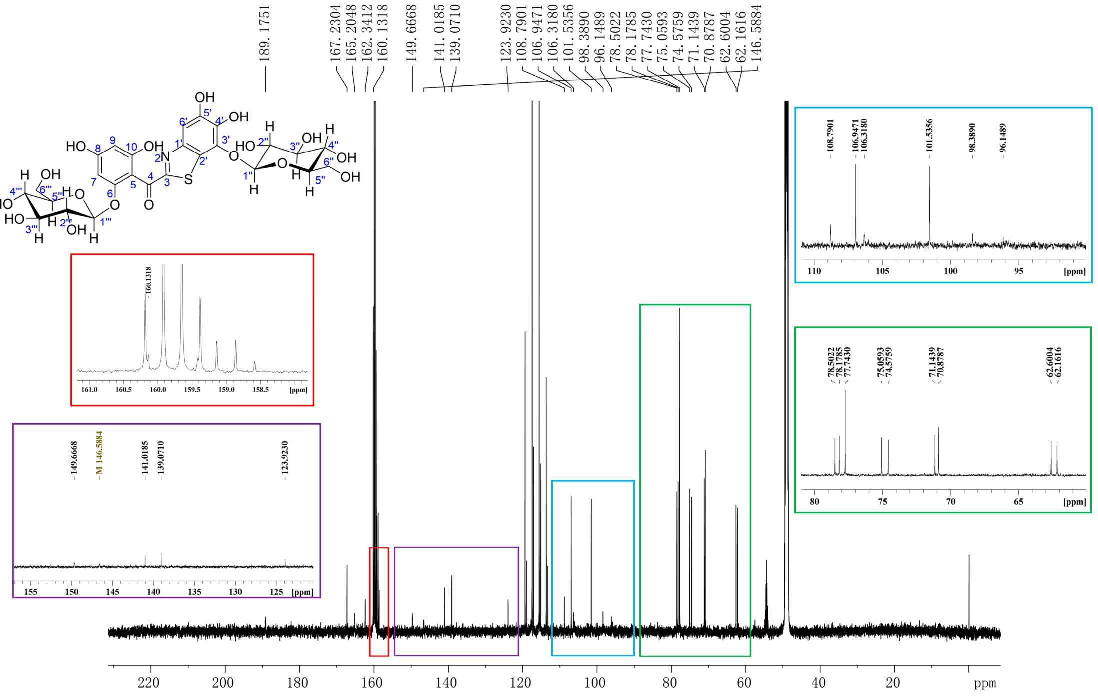

Figure S9. The ^{13}C NMR (150 MHz, CD_3OD:CF_3COOD=20:1) spectrum of ABS-D.

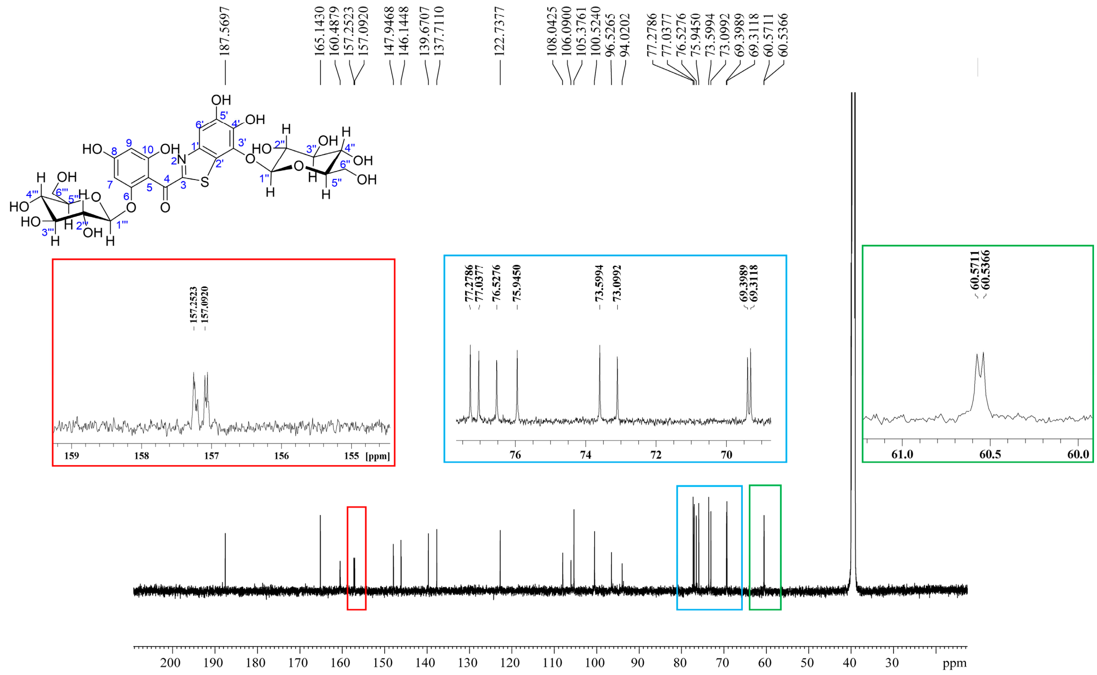

Figure S10. The ^{13}C NMR (150 MHz, DMSO-d_6) spectrum of ABS-D.

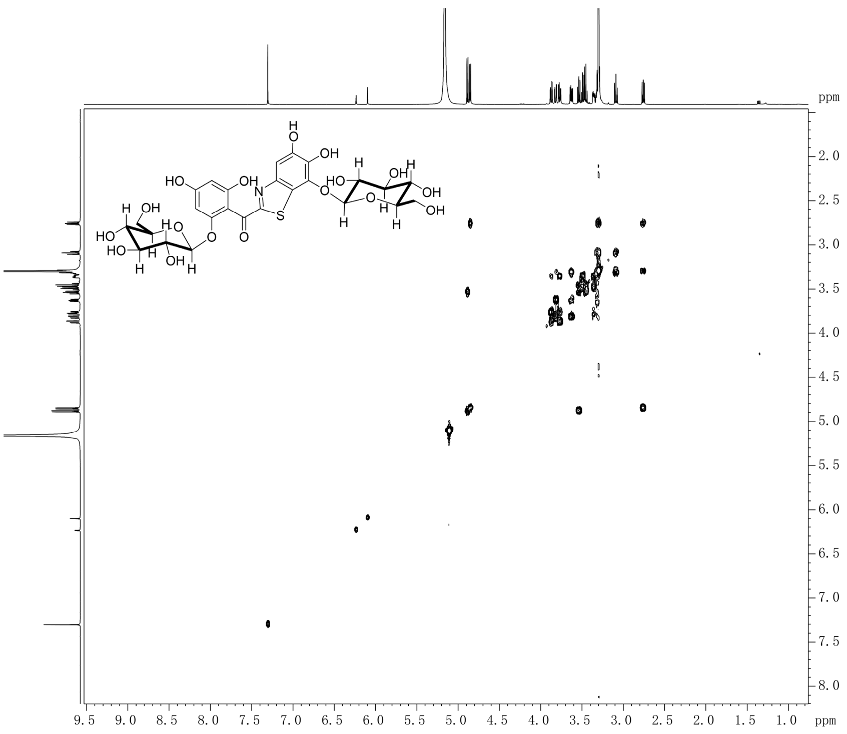

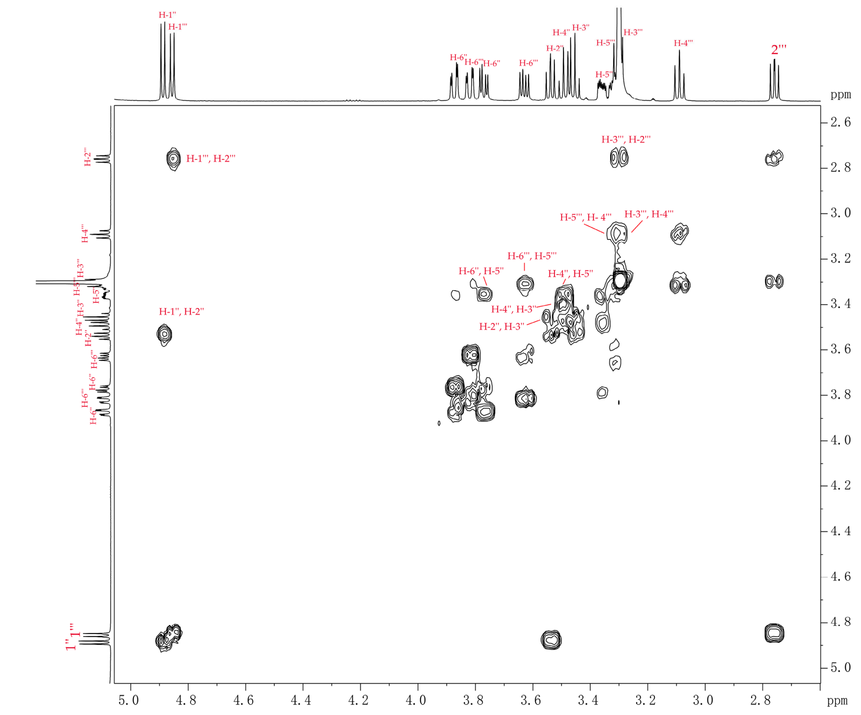

Figure S11. The ^{1}H-^{1}H COSY spectrum of ABS-D in CD_3OD:CF_3COOD=20:1.

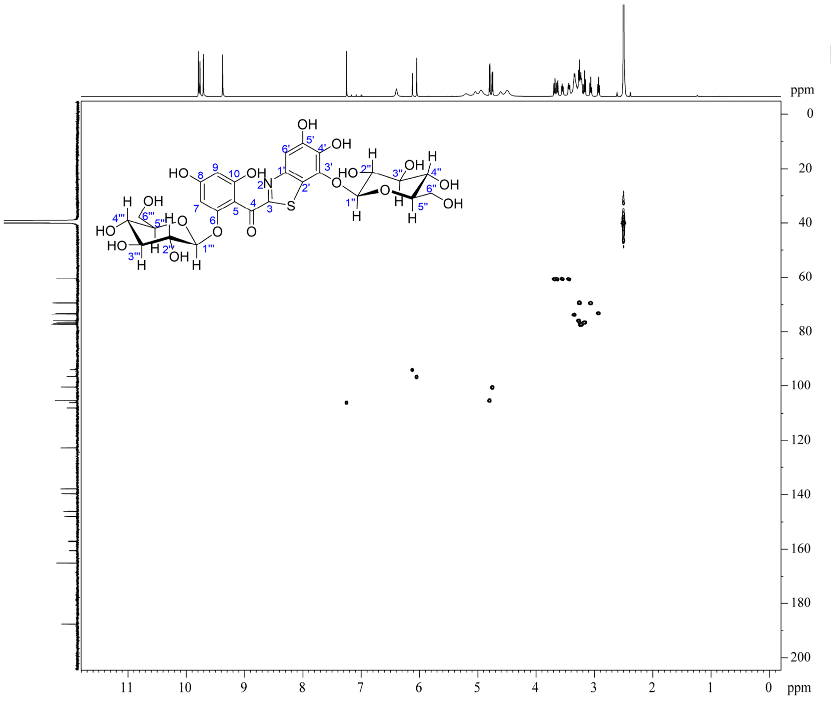

Figure S12. The HSQC spectrum of ABS-D in DMSO-d_6.

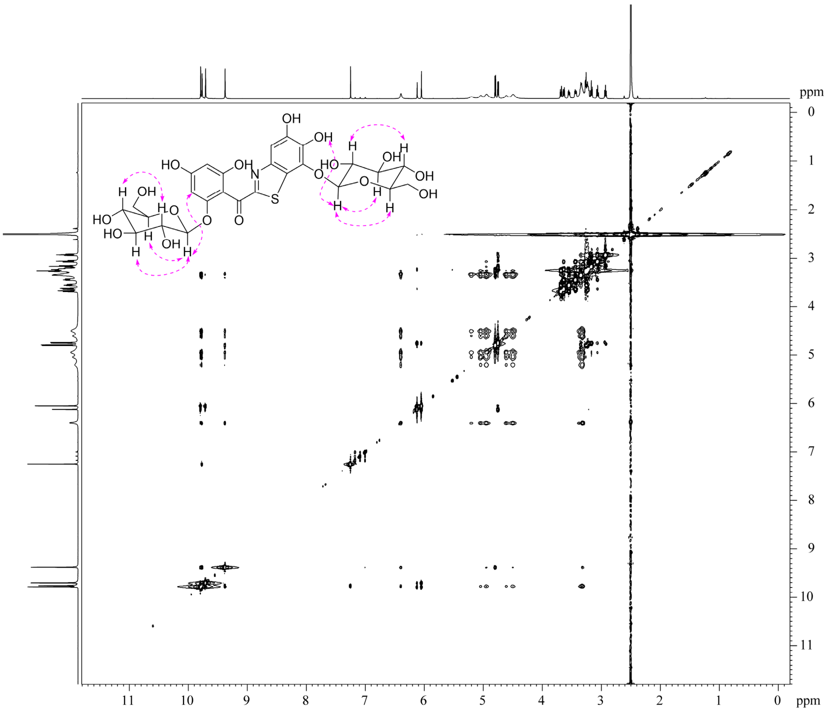

Figure S13. The ROESY spectrum of ABS-D in DMSO-d_6.

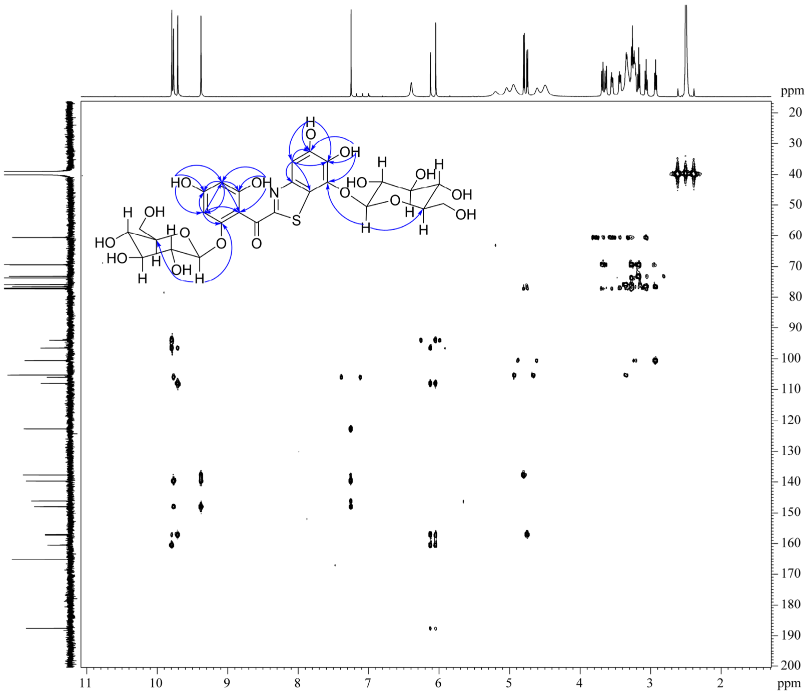

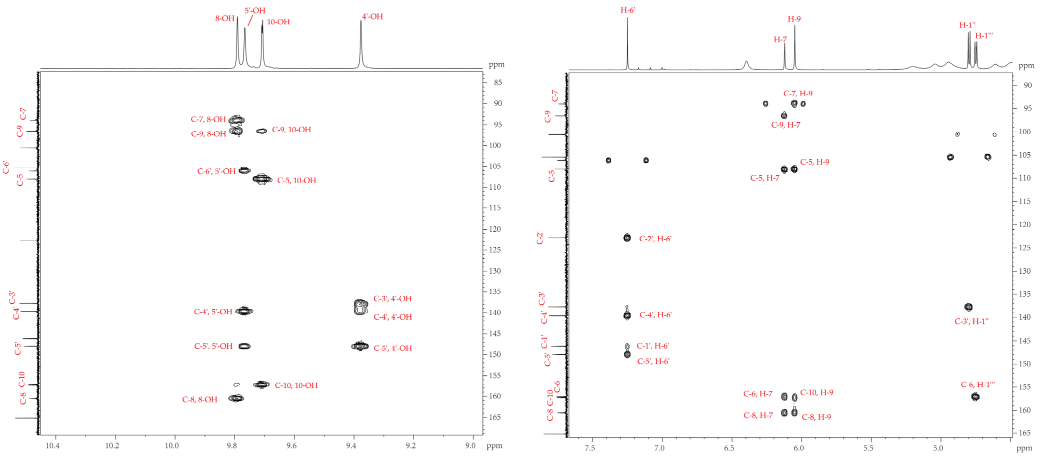

Figure S14. The HMBC spectrum of ABS-D in DMSO-d_6.

Functional		Solvent?		Basis Set		Type of Data	
mPW1PW91		PCM		6-311+G(d,p)		Unscaled Shifts	
		DP4+	99.99%	0.00%	0.01%	–	–
Nuclei	sp2?	xperimenta	Isomer 1	Isomer 2	Isomer 3	Isomer 4	Isomer 5
C	x	165.1	174.6	175.5	170.5		
C	x	187.6	197.1	195.8	199.4		
C	x	108	112.1	114.3	113.3		
C	x	157.1	165.6	166.5	165.6		
C	x	94	101.6	105.5	103.4		
C	x	160.5	171.4	171.7	172.5		
C	x	96.5	101.1	102.3	103.2		
C	x	157.3	174.5	173.5	177.1		
C	x	146.1	150.3	139.3	156.3		
C	x	122.7	135.6	145.6	127.3		
C	x	137.7	144.4	146.0	144.4		
C	x	139.7	148.16	145.31	146.44		
C	x	147.9	151.33	152.80	154.33		
C	x	106.1	110.69	106.93	105.01		
C		105.4	109.70	111.18	107.14		
C		73.6	80.18	79.76	78.25		
C		75.9	84.04	82.61	82.76		
C		69.3	73.80	72.85	72.80		
C		77.3	80.76	81.89	81.41		
C		60.5	63.95	64.00	63.56		
C		100.5	106.76	107.74	108.39		
C		73.1	74.01	73.61	72.94		
C		76.5	83.63	83.26	82.11		
C		69.4	73.32	73.72	72.77		
C		77	82.04	80.77	81.56		
C		60.6	63.8077	61.9255	63.8311		

Figure S15. Experimental and calculated chemical shifts for DP4+ probability analysis for three possible isomers of ABS-D. (see Figure 6A, Isomer 1, 2, and 3 represent structure **a**, **b**, and **c**,

respectively)

Table S1. ^{1}H NMR (600 MHz) and ^{13}C NMR (150 MHz) data of ABS-D in $CD_3OD:CF_3COOD=20:1$

position	δ_H (*J* in Hz)[a]	δ_C
2		
3		167.2
4		189.2
5		108.8
6		160.1
7	6.24, br s	96.1
8		165.2
9	6.10, br s	98.4
10		162.3
1'		146.6
2'		123.9
3'		139.1
4'		141.0
5'		149.7
6'	7.30, s	106.3
1''	4.89, d (7.9)	107.0
2''	3.54, dd (9.0, 7.9)	75.1
3''	3.45, t (9.0)	77.7
4''	3.49 t (9.1)	70.9
5''	3.36, ddd (9.3, 4.6, 2.4)	78.5
6''	3.88, dd (12.1, 2.4)	62.2
	3.77, dd (12.0, 4.7)	
1'''	4.85, d (7.8)	101.5
2'''	2.76, dd (9.2, 7.8)	74.6
3'''	3.30, t (9.2)	77.7
4'''	3.09, t (9.3)	71.1
5'''	3.32, ddd (9.5, 5.9, 2.3)	78.2
6'''	3.82, dd (12.2, 2.3)	62.6
	3.63, dd (12.2, 5.9)	

[a] The indiscernible signals due to overlap or complex multiplicity are reported without designating multiplicity.

Table S2. Calculated and experimental values of ABS-D ^{13}C NMR chemical shifts.

position	δ_{exp}	δ_{cal}		
		**a**	**b**	**c**
3	165.1	174.5968	175.5468	170.4983

4	187.6	197.0714	195.7996	199.3725
5	108	112.0692	114.3085	113.2979
6	157.1	165.5861	166.5191	165.6023
7	94	101.5868	105.5239	103.4206
8	160.5	171.357	171.6536	172.451
9	96.5	101.1236	102.3467	103.1789
10	157.3	174.5486	173.5212	177.0954
1'	146.1	150.295	139.2849	156.2652
2'	122.7	135.5817	145.6144	127.2674
3'	137.7	144.4289	145.9652	144.4458
4'	139.7	148.1582	145.3112	146.4393
5'	147.9	151.3315	152.8004	154.3323
6'	106.1	110.6924	106.9259	105.011
1"	105.4	109.7015	111.1838	107.135
2"	73.6	80.1778	79.7563	78.2528
3"	75.9	84.0411	82.6088	82.7629
4"	69.3	73.8024	72.8469	72.8003
5"	77.3	80.7574	81.8894	81.4101
6"	60.5	63.9476	63.9996	63.5556
1'"	100.5	106.7628	107.7408	108.3932
2'"	73.1	74.005	73.6071	72.9418
3'"	76.5	83.6299	83.2633	82.1063
4'"	69.4	73.3175	73.7202	72.768
5'"	77	82.0405	80.7707	81.5574
6'"	60.6	63.8077	61.9255	63.8311

Table S3. B3-LYP geometries for all the optimized compounds

a

C	4.83099200	-0.93752500	-0.40032500
C	3.47580100	-1.24048100	-0.35000900
C	3.02625100	-2.59728000	-0.29242700
C	4.02306300	-3.62418900	-0.39210000
C	5.38170500	-3.31748900	-0.37763000
C	5.77298100	-1.98118100	-0.37960700
H	5.16185700	0.08015800	-0.55564500
C	1.63171900	-3.01503400	-0.19097000
H	6.11766700	-4.11181600	-0.41671200
C	0.59896000	-2.19210100	0.49653900
C	-0.38464300	-0.96626100	2.04547300
C	-0.50594900	-0.17286800	3.20301700
C	-1.53161600	-1.28672800	1.27877500
C	-1.76488500	0.27070100	3.56413400

C	-2.80063100	-0.81246600	1.63668500
C	-2.91976400	-0.04032800	2.79272000
O	3.69100600	-4.92490800	-0.46814800
O	1.24920600	-4.13457100	-0.59973700
O	7.10318800	-1.72846300	-0.41074000
H	7.25740700	-0.76872300	-0.41767800
O	2.48908200	-0.30578800	-0.45931900
O	-4.13554900	0.40311700	3.19648700
H	-4.00310800	0.89290700	4.02918000
O	-3.92120200	-1.17153100	0.91516700
C	2.70094300	1.09165100	-0.39366300
C	3.21572100	1.61819400	0.96482900
H	1.69196700	1.49596000	-0.57001200
C	3.39174300	3.15161400	0.87182400
H	4.18590500	1.16361800	1.18672700
C	3.67345200	2.88618300	-1.63031400
C	4.23094400	3.54883500	-0.35430500
H	2.67544100	3.30401500	-1.84638900
O	3.55974600	1.46879400	-1.44672000
O	2.37583600	1.24592600	2.03955500
H	1.46963000	1.54290900	1.85209400
C	4.54953100	3.11672400	-2.85136700
H	4.10050400	2.59535200	-3.70880100
H	4.55364400	4.19381500	-3.06342500
O	5.85864200	2.63125000	-2.57804600
H	6.41951500	2.85541000	-3.33648200
C	-4.18134800	-0.42620800	-0.25545700
C	-5.00912900	-1.31206600	-1.19930600
H	-3.23236700	-0.13179100	-0.73375100
C	-5.49782100	-0.49198600	-2.40908200
H	-5.87488000	-1.66480900	-0.62545300
C	-5.22782100	1.57869800	-0.98227400
C	-6.14936700	0.83115900	-1.96784000
H	-4.29988400	1.86801900	-1.50643100
O	-4.90778400	0.72460300	0.12112100
O	-4.29984200	-2.47792100	-1.58659200
H	-3.66357700	-2.23716200	-2.28061800
C	-5.85017100	2.84982800	-0.42607200
H	-5.14191000	3.30347500	0.28249300
H	-5.99667400	3.54623200	-1.26197000
O	-7.08196300	2.52881300	0.20890200
H	-7.51212200	3.36303100	0.45163100
O	-2.04112400	1.03976300	4.66196100
H	-1.23764400	1.19124500	5.18551200

H	2.70859400	-4.96187300	-0.60419200
H	2.39249800	3.59961500	0.76419400
O	3.89884900	3.69971400	2.07706900
H	4.80685700	3.37434500	2.19890100
H	5.26022800	3.20051800	-0.21719400
O	4.33039900	4.95778400	-0.50753400
H	3.43209400	5.32957600	-0.51567400
H	0.37326100	0.06983400	3.79139800
H	-7.09012900	0.61861000	-1.44963400
O	-6.50588000	1.64725700	-3.07522400
H	-5.71301600	1.78738500	-3.62007200
H	-4.62071500	-0.24483100	-3.02726200
O	-6.33033700	-1.25507600	-3.26748900
H	-7.13027900	-1.49961000	-2.77201700
S	-1.08840700	-2.32976400	-0.04838500
N	0.79736800	-1.49223800	1.56974900

b

C	-2.56540300	-0.70490900	1.35552400
C	-1.63621000	-1.49049200	0.68889200
C	-1.26481600	-2.77531700	1.18246400
C	-1.99838300	-3.28897500	2.29955300
C	-2.92656100	-2.49889500	2.97469500
C	-3.18986200	-1.21141700	2.51041500
H	-2.80526500	0.28486900	0.98669100
C	-0.18712100	-3.59112700	0.63640800
H	-3.44983300	-2.89395900	3.83745700
C	1.01874600	-2.95751000	0.03234200
C	3.10909900	-2.52970500	-1.18501400
C	4.28001600	-2.38239400	-1.93623400
C	2.72929000	-1.57548400	-0.21365700
C	5.06242100	-1.26208400	-1.68784500
C	3.53876100	-0.43946400	0.02254000
C	4.70438900	-0.28504100	-0.71626500
O	-1.80071000	-4.53911500	2.75474800
O	-0.16662400	-4.83427500	0.75206000
O	-4.09623800	-0.48042500	3.20051400
H	-4.20722200	0.39018500	2.78234400
O	-1.04288900	-1.09925200	-0.48302000
O	5.51005600	0.78971900	-0.50971700
H	6.26858900	0.70688700	-1.11582400
O	3.22742800	0.45168300	1.02053500
C	-1.73175500	-0.35061900	-1.46633100
C	-3.12601500	-0.91619900	-1.81896100

H	-1.07653300	-0.42759500	-2.34594100
C	-3.86352100	0.02204000	-2.79579800
H	-3.72037000	-0.98755100	-0.90358100
C	-2.36785700	1.88169100	-2.00737100
C	-3.82676400	1.47601800	-2.29134600
H	-1.79642200	1.81700600	-2.94969200
O	-1.79512900	0.99623100	-1.03795200
O	-3.02268700	-2.24454500	-2.29904500
H	-2.59820300	-2.22639600	-3.17354900
C	-2.20911000	3.30276800	-1.48087600
H	-1.14857500	3.57093000	-1.48940900
H	-2.74120400	3.98373400	-2.15003100
O	-2.67596400	3.41977600	-0.13107100
H	-3.45583300	3.99469200	-0.11184700
C	2.07817800	1.26492000	0.84660000
C	1.31402100	1.32058700	2.17400300
H	1.43754200	0.84405200	0.05639500
C	0.13836700	2.30850400	2.03636200
H	2.01602900	1.70908000	2.92969000
C	1.46038200	3.46093500	0.21467700
C	0.60930600	3.66632500	1.48821500
H	0.81761800	3.04463800	-0.57904400
O	2.53348700	2.54539500	0.47359400
O	0.85300000	0.04832200	2.58858300
H	1.04617400	-0.61269100	1.88939900
C	2.06636700	4.74959400	-0.31745600
H	2.65947900	4.51545300	-1.21380000
H	1.24005600	5.40952600	-0.61155600
O	2.87479400	5.34377400	0.69254100
H	3.14094300	6.21996900	0.37434500
O	6.23026200	-0.97740600	-2.33311700
H	6.43257200	-1.65241200	-3.00160400
H	-1.21590100	-4.99798000	2.10036100
H	-3.34710000	-0.01113000	-3.76740800
O	-5.17556900	-0.43402000	-3.07593300
H	-5.68952700	-0.40500100	-2.25111400
H	-4.39419300	1.55592700	-1.35621600
O	-4.45398200	2.37749000	-3.19088100
H	-4.08721800	2.23147400	-4.07929800
H	4.58080900	-3.11417800	-2.67885600
H	1.25394800	4.14890700	2.23304900
O	-0.46290800	4.56430900	1.25192000
H	-1.22496300	4.08245200	0.86294900
H	-0.58042500	1.87416900	1.33044100

O	-0.58643800	2.44589600	3.25283200
H	0.00865900	2.84677800	3.90885300
S	1.92836400	-3.82007400	-1.20983900
N	1.55537900	-1.84737000	0.45118400

c

C	2.39081600	-1.31159300	1.41282900
C	1.38538500	-0.35773300	1.35119200
C	0.46317300	-0.17021700	2.42681600
C	0.59639600	-1.04682500	3.55899800
C	1.63093400	-1.97721100	3.63723600
C	2.51796200	-2.10014600	2.57182800
H	3.03551100	-1.48080500	0.56004900
C	-0.60342100	0.81342200	2.44122100
H	1.72198600	-2.61122100	4.51133400
C	-1.79427600	3.45258900	0.00780900
C	-2.78624800	3.96743400	-0.83529800
C	-1.87707400	2.16057200	0.57776000
C	-3.87765500	3.15773500	-1.10403900
C	-2.97941600	1.33392200	0.23890900
C	-3.98135500	1.83941000	-0.57833900
O	-0.25415600	-0.98945500	4.59109600
O	-1.46286900	0.85580200	3.34627400
O	3.49340300	-3.03122300	2.68330300
H	4.05152300	-3.02329200	1.88715000
O	1.15446800	0.40225800	0.23365200
O	-5.06566000	1.07963300	-0.88304600
H	-5.64186000	1.61093100	-1.46188500
O	-3.12190000	0.05403600	0.73640500
C	2.18638600	0.78347800	-0.66865700
C	3.32218500	1.56638900	0.01846700
H	1.67042000	1.45079900	-1.37258700
C	4.42772100	1.86995800	-1.00573100
H	3.74795000	0.94083000	0.81834000
C	3.61740400	-0.11981600	-2.35269000
C	4.84830200	0.60217000	-1.76656500
H	3.16698400	0.51645400	-3.13308800
O	2.64539000	-0.37214300	-1.32927400
O	2.85509800	2.79928400	0.52610900
H	2.06104800	2.64997300	1.08137300
C	3.95949100	-1.46150200	-2.98100200
H	3.03400700	-1.91781300	-3.36013900
H	4.62952300	-1.27685800	-3.83054500
O	4.57585100	-2.28427100	-1.99668000

H	4.83451500	-3.11428400	-2.42594000
C	-2.34947400	-0.94835100	0.10784100
C	-2.61870400	-2.27455300	0.81375200
H	-1.28185500	-0.68564200	0.14837000
C	-1.83397200	-3.38380400	0.09476000
H	-3.69956100	-2.47243500	0.71859500
C	-1.97616400	-1.99558300	-1.99177400
C	-2.15229900	-3.41547500	-1.40437200
H	-0.90942800	-1.71687000	-1.93203400
O	-2.75655500	-1.05522400	-1.24229200
O	-2.23587100	-2.20758500	2.17391400
H	-2.13132600	-3.13454300	2.45607400
C	-2.39353000	-1.89747300	-3.45030300
H	-2.26139000	-0.85740600	-3.78215300
H	-1.71782600	-2.53509800	-4.03527600
O	-3.74765800	-2.31355900	-3.57994300
H	-3.95223900	-2.35296400	-4.52680300
O	-4.93029300	3.51215900	-1.89309700
H	-4.81453700	4.41059800	-2.24392800
H	-0.94743600	-0.31273500	4.34883900
H	4.02032700	2.59480200	-1.72423900
O	5.53379500	2.53325700	-0.41363300
H	5.95226200	1.91839700	0.21254800
H	5.34643200	-0.08707000	-1.07663300
O	5.80708100	0.87791200	-2.77855800
H	5.45920000	1.58902800	-3.34285000
H	-2.71547900	4.96408700	-1.25956000
H	-3.19467000	-3.71456200	-1.55530400
O	-1.37844400	-4.37650400	-2.10836900
H	-0.43901100	-4.20828800	-1.92327700
H	-0.76379700	-3.17169200	0.22596900
O	-2.03025900	-4.62807400	0.76128600
H	-2.93930500	-4.92720500	0.58518000
S	-0.32954600	4.22624100	0.55447700
N	0.14199300	2.90650400	1.51497500
C	-0.73833100	1.93638500	1.43581600

TMS

Si	0.00017900	-0.00011900	-0.00004300
C	-0.94550700	-1.43927300	-0.78963900
H	-1.96310600	-1.14165200	-1.07558800
H	-0.44202900	-1.80203200	-1.69558600
H	-1.03295300	-2.28868800	-0.09921000
C	-0.91820600	0.58306600	1.55087500

H	-1.00686300	-0.22220900	2.29218200
H	-0.39709500	1.41976100	2.03493600
H	-1.93456900	0.92294200	1.31143900
C	0.12167300	1.42999500	-1.23647200
H	0.66349300	2.28457800	-0.80994600
H	0.65090600	1.12730100	-2.14980000
H	-0.87277100	1.78701100	-1.53565700
C	1.74198300	-0.57374000	0.47530600
H	1.70931200	-1.40291500	1.19447900
H	2.30311300	-0.92121300	-0.40232700
H	2.32038800	0.23848300	0.93526700
